# Linearity of a Silicon Photodiode at 30 MHz and Its Effect on Heterodyne Measurements

**DOI:** 10.6028/jres.096.005

**Published:** 1991

**Authors:** Alan L. Migdall, Carsten Winnewisser

**Affiliations:** National Institute of Standards and Technology, Gaithersburg, MD 20899

**Keywords:** heterodyne, high frequency, irradiance, linearity, photodiode, saturation

## Abstract

The effect of optical irradiance on the linearity of a Si photodiode was studied. These results are compared for light modulated at 30 MHz and at dc as the optical irradiance was varied over a 9 decade range. We discuss how these results affect the use of this detector as a heterodyne receiver. As the optical irradiance varied from 10^−2^ to 10^+3^ mW/cm^2^, while maintaining constant total power, the photocurrent was constant to about 1%, but as the power density increased further, the photocurrent increased about 13%. At the highest densities that we could achieve, about 6×10^7^ mW/cm^2^ there was only slight evidence of the onset of saturation. These results are of importance in our work to use optical heterodyne detection to measure filter transmittances over a wide dynamic range. The results provide guidelines for achieving maximum accuracy when using this particular diode as an optical heterodyne receiver.

## 1. Introduction

Recently [1,2] we showed how optical heterodyne detection, as proposed by Snyder [3], can be used to measure filter transmittances over a very wide dynamic range and to tie optical transmittance to rf attenuation standards in an absolute way. The accuracy of that technique depends on the linearity of the heterodyne receiver at the heterodyne frequency, 30 MHz in our case. We measured the linearity of the heterodyne receiver, a Si photodiode, used in those filter transmittance measurements as the optical power density was varied over 9 decades. These results are used to define the useful range of operating conditions of that detector for our heterodyne application. These results are compared to previous high-frequency linearity measurements of a different detector by Young and Lawton [4].

## 2. Experiment

To determine how detector responsivity varies with optical irradiance, the detector photocurrent was recorded as the detector moved through the focus of a laser beam. Since the total power on the detector was constant, any change in the photocurrent indicates a variation in detector response with optical irradiance. This method was used to measure both the 30 MHz and dc responsivities. This technique depends on good uniformity of response over the detector surface. The nonuniformity was found to be less than 1%, which was significantly smaller than the irradiance dependent variations of responsivity that were of interest.

Our source of light with a 30 MHz modulation was the output beam of the Mach-Zender interferometer used for our heterodyne measurements of filter transmittance described in reference [1]. That setup used a He-Ne laser at 632.8 nm as the light source for the interferometer. The interferometer contained an acousto-optic modulator (AOM) in each of the two optical paths to shift the frequency of each of the light beams. The AOMs were operated with different drive frequencies to produce a 30 MHz frequency difference between the two light beams. These two beams were recombined at the output of the interferometer producing an optical beam modulated at the 30 MHz difference frequency. We used this modulated output beam as our source and focused it using a 25 mm focal length achromatic lens.

A beam profiler was used to measure the spatial profile of the output beam from which the peak power density was calculated. The beam profiler, a Photon, Inc.^2^ model 1180-14, works by scanning a 50 μm slit across a Si photodiode. This device reads out the beam diameter at a selected fraction of the maximum signal level. This system works well for half maximum beam diameters of at least twice the 50 μm slit size. To determine the actual diameters of beams smaller than this, we recorded the apparent beam diameters at 25% and 75% of the peak level. The difference between these two diameters was used to determine the true 50% beam diameter. The functional dependence of the measured 25%–75% diameter difference on the actual 50% diameter was calculated in a straight-forward manner assuming a gaussian beam shape. This method is a sensitive means of determining the size of very small beams.

We verified the beam diameter measurement technique and the quality of the beam itself by determining the diameter of the beam as a function of position along the beam. The data were found to be well modeled by a gaussian beam with a 3.4 μm 50% diameter at the waist. We used the beam diameters in only one dimension, even though the beam was somewhat elliptical in cross section (about 25% difference in horizontal and vertical diameters). The error introduced into the irradiance calculations by this simplification was small compared to the 9 decade range of the measurements.

**Figure 1:**
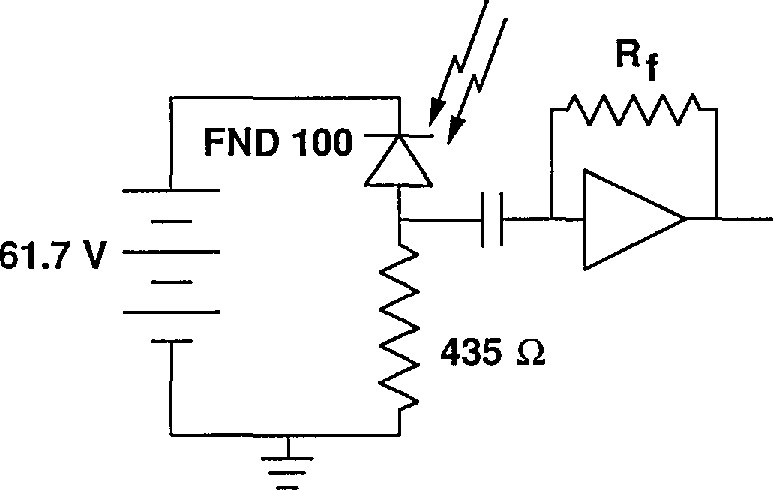
Detector circuit.

The optical heterodyne detector was a windowless EG&G FNDIOO PIN type Si photodiode reverse biased with 61.7 V through a 435 Ω resistor (see fig. 1). The voltage drop across the resistor of −0.2 V was always very much smaller than the drop across the diode so the diode bias remained nearly unchanged. This insured that our detection circuit did not contribute to any nonlinearity. The 30 MHz signal was ac coupled to the input of a transimpendence amplifier. The bias resistor was made as large as practically possible relative to the capacitor impedence to reduce the shunt to ground of the ac signal. The magnitude of the 30 MHz signal was measured with 0.001 dB resolution using a modified signal and attenuation calibrator [2].

The dc photocurrent was determined by measuring the voltage drop across the 435 ft resistor. To estimate the absolute internal quantum efficiency of this photodiode at dc and for low optical irradiance, we compared its response at 632.8 nm to a laser power meter that we calibrated against a 99.6 ± 0.1% efficient multi-reflection Si photodiode “trap” device [5]. Also required for this estimate was the reflectivity of the FNDIOO, which we measured to be 16%. The internal quantum efficiency of the diode at dc was found to be 0.87 ± 0.01.

## 3. Results

To determine the FNDIOO detector response, the 30 MHz signal was recorded as the detector was moved through the focus. The single maximum in the output signal was the reference point that allowed us to match up the positions of the detector to the positions at which the beam diameter was measured. Figure 2a shows the relative response of the detector to a 30 MHz signal as a function of the optical irradiance of the peak of the spatial distribution at the detector surface. Four different data sets are shown. The points indicated the diamonds were taken at full laser power. The other sets were taken with attenuators in the laser beam to allow the range of peak irradiances to be extended. The four data sets were shifted vertically to produce a continuous curve and the flat region of the combined curve was chosen arbitrarily as a response of 1. That flat region is where the detector response is linear. The response changed by less than 1% up to an irradiance level of 10^3^ mW/cm^2^. Above this level the detector exhibits an increasing responsivity, or supralinearity, of up to 12%. At the highest irradiance, above 10^6^ mW/cm^2^, there is some indication of the onset of saturation.

For comparison purposes, we also measured the responsivity of the detector versus irradiance at dc. Figure 2b shows the relative response of the diode at dc as a function of the peak optical irradiance at the detector surface. Three different data sets are shown. The triangles and diamonds indicate distinct data sets taken at different total optical powers. The circles indicate a data set taken later to check the repeatability of the measurement. The sensitivity of our dc voltmeter did not allow us to follow the responsivity curve to irradiances as low as those reached with the 30 MHz data but we were able to take enough data to observe the linear region. As before, the response in the flat region was chosen arbitrarily to be 1. Both the dc and 30 MHz responsivities show the same irradiance threshold for the onset of nonlinearity and approximately the same rate of increase above the threshold.

Since no internal gain is expected, the largest the internal quantum efficiency of our photodiode can be is 1. As a result the low-irradiance dc quantum efficiency of 0.87 can rise by at most 15%. The total measured increase of the dc responsivity is 12.5%, nearly the same as for the 30 MHz data. This level of increase is nearly equal to the maximum allowable increase.

## 4. Conclusions

For the FND 100 diode used in our heterodyne measurements, the dependences of the 30 MHz and dc responsivities on irradiance were nearly the same. The detector was linear at irradiance levels below 10^3^ mW/cm^2^. At irradiance levels above 10^3^ mW/cm^2^ the response increased by up to 13%. This brought the internal quantum efficiency to nearly 100% as determined by the absolute measurement of the low-irradiance internal dc quantum efficiency.

This level of increase in responsivity can be explained by the saturation of loss mechanisms such as recombination at trap sites [6]. Impurities within the diode act as trap sites where photogenerated charge carriers can be caught long enough to recombine. This reduces the quantum efficiency to less than 1. As the optical irradiance and thus the carrier concentration increases, the trap sites fill up. This reduced loss has the effect of increasing the quantum efficiency up toward a maximum level of 1, although other saturation mechanisms may become important before that limit is reached. The quantum efficiency increased nearly to 1 with only a hint of responsivity saturation at the highest irradiances that we could achieve.

The results here are in contrast to the work of Young et al. that compared the dc and 600 MHz responsivities of a HP 5082-4220 PIN type diode. They saw no evidence of supralinearity in either the dc or high-frequency responsivities. They also found differences between the two responsivities that our FND 100 diode did not exhibit.

As a result of these measurements, we have found that our detector/amplifier package can be used to make linear measurements of an optical heterodyne signal as long as the irradiance is less than 10^3^ mW/cm^2^. Even at irradiances larger than this, the optical heterodyne measurements can still be made linear, as long as the local oscillator beam remains constant and is significantly more powerful than the signal beam. If this is the case, the responsivity remains nearly constant, because as the signal beam irradiance changes, the total irradiance does not vary much. With these easy to achieve conditions, our heterodyne receiver is linear and can be used in high-accuracy applications.

## Figures and Tables

**Figure 2a:**
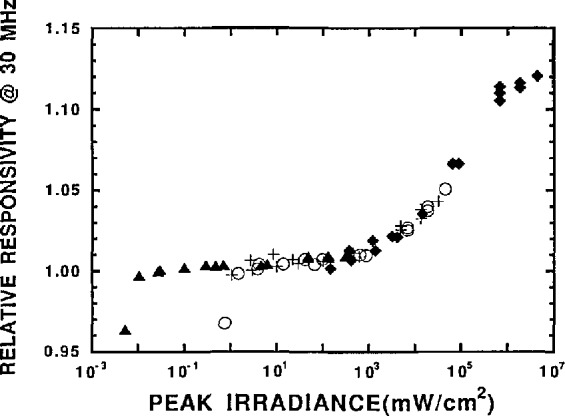
Relative detector response at 30 MHz versus peak optical irradiance on the detector. The different symbols indicate data taken at different total optical powers. The different data sets were shifted to produce a continuous curve. The response in the flat region was arbitrarily chosen to be 1. The two low points were due to overfilling the detector.

**Figure 2b:**
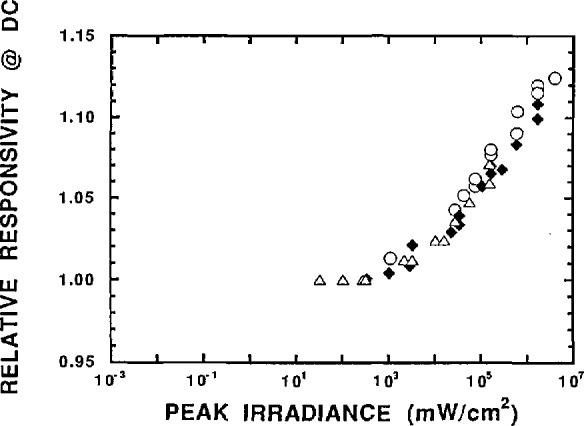
Relative detector response at dc versus peak optical irradiance on the detector. The response in the flat region was arbitrarily chosen to be 1. The triangles and diamonds indicate distinct data sets taken at different total optical powers. The circles Indicate a data set taken at a later time to check the repeatability.
